# PUBLIC MENTAL HEALTH SERVICE USE AMONG US ADULTS AGE 50+ COMPARED TO YOUNGER AGE GROUPS

**DOI:** 10.1093/geroni/igac059.2146

**Published:** 2022-12-20

**Authors:** Namkee Choi, Diana DiNitto

**Affiliations:** University of Texas at Austin, Austin, Texas, United States; University of Texas at Austin, Austin, Texas, United States

## Abstract

Despite increasing numbers of older-adult mental health service users, few studies have examined their use of public mental health services. Using the 2018 and 2019 Mental Health-Client Level data (N=4,291,737 in 2018 and N=4,513,946 in 2019 for those age 18+), we examined age group differences in the types of mental disorders diagnosed in outpatient-only, both outpatient and inpatient, and inpatient-only service settings. Of all users, 25.3% were age 50-64 and 6.7% were age 65+. Multivariable logistic regression results, controlling for gender, race/ethnicity, census region, and alcohol/substance use disorder, showed that compared to the 30-49 age group, the 50-64 and 65+ age groups had higher odds of having depressive disorder in outpatient-only settings (aOR=1.28 [95% CI=1.28-1.29] and aOR=1.08 [95% CI=1.08-1.09] for the 50-64 and 65+ age groups, respectively). Both older groups also had higher odds of delirium/dementia disorder in all three service settings. In addition, they had consistently higher odds of a diagnosis of schizophrenia or other psychotic disorder in all three service settings (aOR=1.88 [95% CI=1.86-1.89], aOR=1.70 [95% CI=1.65-1.74], and aOR=1.44 [95% CI=1.39-1.49] in outpatient, both outpatient and inpatient, and inpatient-only settings, respectively). Community mental health centers (CMHC) are on the frontlines in serving vulnerable communities and received increased federal funding during the COVID-19 pandemic. Our findings indicate that CMHCs also need programs dedicated to and tailored for older adults. More research is also needed on older adults who receive public mental health services and unmet mental health needs among low-income older adults with serious mental illness.

